# Cross-Phosphorylation between AgrC Histidine Kinase and the Noncognate Response Regulator Lmo1172 in *Listeria monocytogenes* under Benzalkonium Chloride Stress

**DOI:** 10.3390/microorganisms12020392

**Published:** 2024-02-16

**Authors:** Tao Yu, Xiaojie Jiang, Xiaobo Xu, Ping Xu, Shuxing Qiu, Junlei Yin, David P. Hamilton, Xiaobing Jiang

**Affiliations:** 1 School of Biological Engineering, Xinxiang University, Xinxiang 453003, China; yutao@xxu.edu.cn (T.Y.); jiangxiaojie1987@hotmail.com (X.J.); xuxb0423@163.com (X.X.); suyongyu@126.com (P.X.); 2 Australian Rivers Institute, Griffith University, Brisbane, QLD 4111, Australia; david.p.hamilton@griffith.edu.au; 3 Key Laboratory of Biomedicine and Health Risk Warning of Xinxiang City, Medical College, Xinxiang University, Xinxiang 453003, China; qsx04152006@163.com (S.Q.); lyin22@163.com (J.Y.); 4 Henan Engineering Laboratory for Bioconversion Technology of Functional Microbes, College of Life Sciences, Henan Normal University, Xinxiang 453007, China

**Keywords:** *Listeria monocytogenes*, AgrC, Lmo1172, crosstalk, benzalkonium chloride

## Abstract

Benzalkonium chloride (BC) is widely used for disinfection in the food industry. However, *Listeria monocytogenes* strains with resistance to BC have been reported recently. In *L. monocytogenes*, the Agr communication system consists of a membrane-bound peptidase AgrB, a precursor peptide AgrD, a histidine kinase (HK) AgrC, and a response regulator (RR) AgrA. Our previous study showed that the *agr* genes are significantly upregulated by BC adaptation. This study aimed to investigate the role of the Agr system in BC resistance in *L. monocytogenes*. Our results showed that the Agr system was involved in BC resistance. However, a direct interaction between BC and AgrC was not observed, nor between BC and AgrA. These results indicated that BC could induce the Agr system via an indirect action. Both AgrBD and AgrC were required for growth under BC stress. Nevertheless, when exposed to BC, the gene deletion mutant ∆*agrA* strain exhibited better growth performance than its parental strain. The RR Lmo1172 played a role in BC resistance in the ∆*agrA* strain, suggesting that Lmo1172 may be an alternative to AgrA in the phosphotransfer pathway. Phosphorylation of Lmo1172 by AgrC was observed in vitro. The cognate HK Lmo1173 of Lmo1172 was not involved in BC stress, regardless of whether it was as the wild-type or the ∆*agrA* mutant strain. Our evidence suggests that the HK AgrC cross-phosphorylates its noncognate RR Lmo1172 to cope with BC stress when the cognate RR AgrA is absent. In vivo, further studies will be required to detect phosphotransfer of AgrC/AgrA and AgrC/Lmo1172.

## 1. Introduction

*Listeria monocytogenes* is a Gram-positive bacterial pathogen that is ubiquitously distributed in natural environments [1]. It can cause a severe infectious disease, listeriosis, which occurs in humans and animals, with a mortality of up to 20 to 30% [2,3]. *L. monocytogenes* is frequently detected in various types of food, such as raw meat, dairy products, eggs, and ready-to-eat foods, establishing its significance as an important foodborne pathogen [4,5,6]. *L. monocytogenes* exhibits the capacity to persist in food processing environments for prolonged durations, thereby elevating the risk of food contamination caused by this pathogen [7,8].

Microbial food safety is dependent on disinfection in the food industry. Benzalkonium chloride (BC), which belongs to quaternary ammonium disinfectants, is widely used in food processing environments for microorganism control due to its excellent antibacterial activity against many important foodborne pathogens [9,10]. However, the frequent use of BC has facilitated the emergence of resistant isolates. Up to now, the low sensitivity to BC of *L. monocytogenes* strains from foods and food environments has been reported in different countries [9,11,12]. In most cases, the efflux pump that is located on the chromosome and/or mobile genetic element is responsible for BC resistance in *L. monocytogenes* [13,14,15].

The Agr system, a peptide-mediated communication system that was first described in *Staphylococcus aureus*, has been identified in *L. monocytogenes* [16]. The *agr* locus of *L. monocytogenes* comprises the *agrBDCA* operon, encompassing four genes. These genes encode the histidine kinase (HK) AgrC and the response regulator (RR) AgrA of a two-component signal transduction system, as well as a membrane-bound peptidase AgrB, which is involved in the processing of a precursor peptide AgrD into an autoinducing peptide (AIP) and its exportation [16,17,18]. The Agr system plays an important role in many physiological processes in *L. monocytogenes*, such as biofilm formation, virulence, and invasion [16,19,20]. Our previous study has reported that the *agr* operon is significantly upregulated by BC adaptation, indicating that the Agr system is associated with BC resistance in *L. monocytogenes* [21]. 

This study aimed to investigate the involvement of the Agr system and its components in conferring BC resistance in *L. monocytogenes*. 

## 2. Materials and Methods

### 2.1. Strains, Plasmids, and Media

All strains and plasmids are listed in Supplementary Table S1. *L. monocytogenes* strains were cultured using brain heart infusion (BHI; Oxoid Ltd., Basingstoke, Hampshire, UK) broth or BHI agar. *Escherichia coli* strains were cultured in Luria–Bertani (LB) broth or agar. The primers utilized are presented in Supplementary Table S2.

### 2.2. Determination of MIC

BC was obtained from Shanghai Macklin Biochemical Co., Ltd. (Shanghai, China). The agar dilution method was performed to determine MICs of BC against *L. monocytogenes* strains as described previously [22]. Briefly, bacterial cultures were diluted in sterilized saline solution, and an inoculum of 10^4^ to 10^5^ cells per ml was plated on Mueller–Hinton agar (MHA; Huankai Ltd., Guangzhou, China) supplemented with 2% defibrinated sheep blood. MICs were recorded after 24 h of incubation at 37 °C. 

### 2.3. Determination of Gene Transcription Levels

qRT-PCR analysis of gene transcription levels was carried out according to the method of Jiang et al. [23]. To investigate the effects of BC on gene transcription levels, the *L. monocytogenes* strain was grown to a logarithmic growth phase (OD_600_ of 0.6). Half of the culture was exposed to 2 μg/mL of BC for 30 min, and the other half was further grown in BHI broth without BC for 30 min. To determine the relative gene transcription levels in the mutant strain, the wild-type strain EGD-e and the mutant strain were incubated to the logarithmic growth phase. Then, samples were subjected to total RNA extraction, reverse transcription reaction, and qPCR. To ensure the amplification efficiencies of primers between 90 and 110%, qPCR conditions were optimized by adjusting the annealing temperatures and primer concentrations. 16S rRNA was used as a reference gene. Relative transcription levels were calculated using the 2^−ΔΔCt^ method [24].

### 2.4. Analysis of the agr Promoter Activity

The *agr* promoter (P_2_)-*lac*Z fusion was constructed following previously described methods [25,26]. The P_2_ was inserted into the plasmid pPTPL (a low-copy-number promoter probe vector), and the recombinant vector was finally electroporated into EGD-e or ∆*lmo1172*. To investigate the effects of BC on the P_2_ activity, the culture of EGD-epPTPL-P_2_ in the logarithmic growth phase was exposed to different concentrations of BC (1 μg/mL and 2 μg/mL) for 30 min. Bacterial culture without treatment of BC was used as a control. To compare the P_2_ activity in the wild-type EGD-e and the ∆*lmo1172* strain, EGD-epPTPL-P_2_ and ∆*lmo1172*pPTPL-P_2_ were incubated to the logarithmic growth phase. *β*-galactosidase activity assay was then carried out according to the method described by Miller [27]. 

### 2.5. Deletion Mutant Construction

Nonpolar deletion mutants were constructed from the parent strain EGD-e as described previously [28]. The temperature-sensitive pMAD shuttle vector was used for generating mutants. An insert containing homologous arms up- and down-stream of the target gene was obtained by splicing by overlap extension PCR. The insert was cloned into pMAD and transformed into *E. coli* DH5α. After confirmation by sequencing, the recombinant vector was electroporated into the competent *L. monocytogenes* cells. Single-cross-over mutants were selected at 39 °C with erythromycin (5 μg/mL) to promote chromosomal integration. Double-cross-over mutants were selected at 39 °C without antimicrobial to enable plasmid excision and curing. All deletion mutants were confirmed by PCR.

### 2.6. Complementation

Complementation experiments were performed using pERL3 [29] as described previously [30]. The promoter in pERL3 had no effect on the expression of down-stream genes. Sequences used to construct complemented strains, including the P_2_ promoter and the *agr* operon, the P_2_ promoter and *agrBD*, and the up-stream region and coding sequence of *lmo1172*, were amplified by PCR. The sequence containing P_2_ and *agrC* was synthesized by GenScript (Nanjing, Jiangsu, China). After digestion with appropriate enzymes, the target gene was cloned into pERL3, and the recombinant plasmid was then electroporated into *L. monocytogenes* competent cells. Plasmid-containing cells were selected on BHI agar plates with erythromycin (10 μg/mL). 

### 2.7. Growth Curve Analyses

Growth curve analyses of *L. monocytogenes* strains were performed as described by Jiang et al. [31] using a Bioscreen C microbiology reader (Helsinki, Finland). Briefly, five colonies of each strain were individually inoculated into 5 mL of BHI broth and incubated overnight at 37 °C. The cultures were diluted in fresh BHI broth with or without BC. Three hundred microliters of each suspension was transferred into 100-well plate. The strains were grown in a Bioscreen C microbiology reader at 37 °C, and the OD_600_ value was measured at 15 min intervals. 

### 2.8. Preparation of Recombinant Proteins

The full-length proteins of AgrC and AgrA were obtained in our previous study [32]. Lmo1172 and AgrA_(150–242)_ (the DNA-binding domain of AgrA) were expressed and purified as described previously [22]. Briefly, the target gene was amplified and cloned into pET-28a expression vector. Recombinant protein was expressed in *E. coli* BL21 (DE3) and purified using BeyoGold His-tag purification resin (Beyotime Biotechnology Co., Shanghai, China). Expression and purification of Lmo1173 were conducted following previously established procedures [33]. The full-length open reading frame of *lmo1173* was cloned into pET-28a and then introduced into *E. coli* C43 (DE3) for further processing. Induced expression of Lmo1173 was conducted by adding isopropyl *β*-D-1-thiogalactopyranoside (IPTG). Immobilized metal affinity chromatography and size exclusion chromatography were employed to purify Lmo1173. SDS-PAGE was performed to detect the purified proteins.

### 2.9. In Vitro Phosphorylation Assays

The reactions of HK autophosphorylation and HK/RR phosphorelay were examined as described previously [34]. The autophosphorylation reaction was conducted as follows: purified HK (0.5 μg) was incubated in the reaction buffer [0.3 M Tris–HCl (pH 8.0) containing 50 mM KCl, 10 mM MgCl_2_, and 10 mM ATP] for 1 min at 25 °C. The phosphotransfer reaction was conducted as follows: the HK protein alone was incubated with ATP for 1 min. Then, the RR protein was added and incubated for one more minute. All reactions were stopped by the addition of SDS loading buffer. Samples were analyzed by Mn^2+^-Phos-tag SDS-PAGE. Unlike normal SDS-PAGE gel, Mn^2+^-Phos-tag SDS-PAGE gel was prepared by adding the acrylamide-pendent Phos-tag ligand (Nard Institute Ltd., Amagasaki, Japan) and MnCl_2_ to the separating gel. After electrophoresis, the gel was stained with CBB G-250 (Sangon Biotech, Shanghai, China), and the image was acquired using the ChemiDoc XRS+ imaging system (Bio-Rad, Hercules, CA, USA). In the autophosphorylation and phosphotransfer reactions, AgrA_(150–242)_, the DNA-binding domain that cannot be phosphorylated, was used as the negative control. The phosphate groups of phosphorylated proteins bound to metal ions in Phos-tag during the electrophoresis process, resulting in a slower migration rate. The relative migration rates of the interest proteins (*R_f_*) were calculated and compared to distinguish between phosphorylated and non-phosphorylated proteins. The *R_f_* value was equal to the ratio of protein migration distance to bromphenol blue dye migration distance. 

### 2.10. MST

Proteins were labeled with fluorescence using the Protein Labeling Kit (NanoTemper Technologies, Munich, Germany) following the manufacturer’s instructions. Compound BC was diluted as a gradient through the capillaries with the protein. Measurements were performed using a Monolith NT.115 apparatus (NanoTemper). 

### 2.11. Detection of Lmo1173 Kinase Activity

Kinase-Lumi Chemiluminescence kinase activity detection kit (Beyotime) was used to measure the kinase activity of Lmo1173 according to the manufacturer’s instructions. Briefly, the purified protein was incubated with the reaction buffer for 5 min at 37 °C, and then, ATP was added. After incubation for 10 min, the chemiluminescence of the samples was detected using a CLARIOstar multimode microplate reader (BMG Labtech, Offenburg, Germany).

### 2.12. EMSA

DNA–protein binding was set up in reactions containing 200 ng of DNA, binding buffer (Beyotime), and purified recombinant protein. DNA–protein complex was separated by 6% non-denaturing polyacrylamide gel electrophoresis, and its visualization was achieved using ethidium bromide staining. 

### 2.13. Statistical Analysis

Statistical analysis of the results was conducted using analysis of variance (ANOVA). Results with a calculated *p* value < 0.05 were considered statistically significant.

## 3. Results

### 3.1. The Presence of BC Induces Transcription of the agr Operon

**Figure 1:**
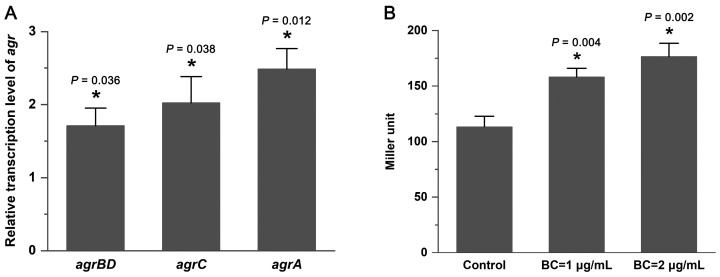
BC induces transcription of the *agr* operon in *L. monocytogenes*. (**A**) Relative transcription levels of *agr* genes in the presence of BC (2 μg/mL) were determined by qRT-PCR. The results are presented as fold changes relative to the transcription level of the target gene in *L. monocytogenes* EGD-e without BC. (**B**) Effects of BC on the activity of the *agr* promoter (P_2_). Error bars represent the standard deviation of triplicate experiments (*n* = 3). The asterisk indicates a value that is statistically different from that of the control at *p* < 0.05.

The minimum inhibitory concentration (MIC) of BC against *L. monocytogenes* EGD-e was 6 μg/mL. Quantitative real-time PCR (qRT-PCR) was conducted to determine the relative transcription levels of *agrBDCA* in the presence of BC (2 μg/mL). The data showed that *agrBD*, *agrC*, and *agrA* were significantly upregulated (*p* < 0.05) by BC (Figure 1A). In addition, the effects of BC on the activity of the *agr* promoter P_2_ were also investigated. BC at 1 μg/mL and 2 μg/mL increased the P_2_ activity by 40% and 56%, respectively (Figure 1B). These findings indicate that BC induces the transcription of the *agr* operon. 

### 3.2. The Agr System Is Involved in BC Resistance

**Figure 2:**
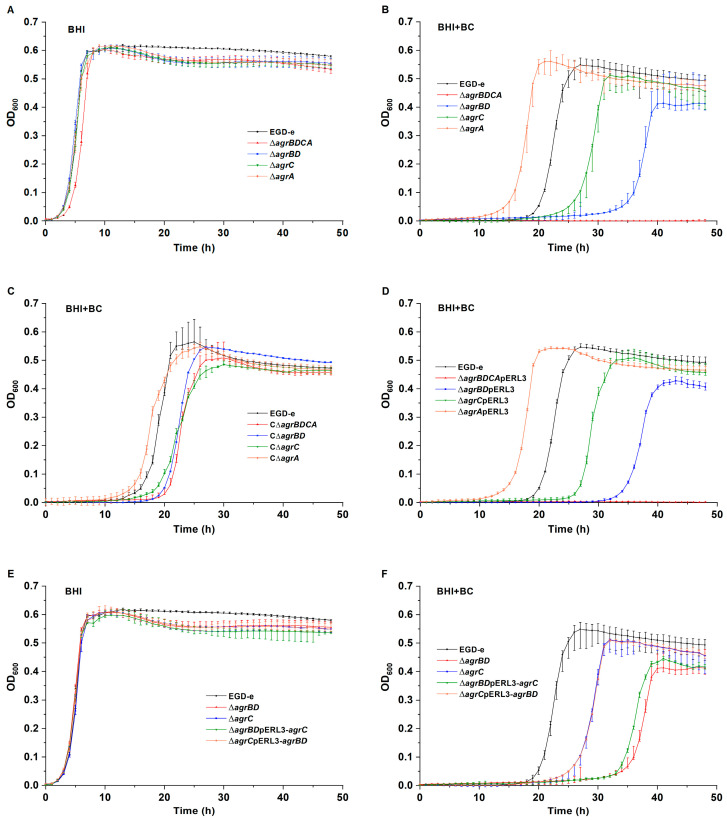
Growth curves for *L. monocytogenes* wild-type EGD-e, ∆*agrBDCA*, ∆*agrBD*, ∆*agrC*, and ∆*agrA* strains in (**A**) BHI broth and (**B**) BHI broth with 2 μg/mL of BC. (**C**) Growth curves for EGD-e, the C∆*agrBDCA*, C∆*agrBD*, C∆*agrC*, and C∆*agrA* complemented strains in BHI broth with 2 μg/mL of BC. (**D**) Growth curves for the vector controls in BHI with 2 μg/mL of BC. Growth curves for EGD-e, ∆*agrBD*pERL3-*agrC*, and ∆*agrC*pERL3-*agrBD* in (**E**) BHI broth and (**F**) BHI broth with 2 μg/mL of BC.

Based on the results mentioned above, it was speculated that the Agr system could be associated with BC resistance. The mutant lacking the entire *agr* operon was constructed to confirm this hypothesis. The gene deletion mutant ∆*agrBDCA* demonstrated an identical MIC for BC to the wild-type strain EGD-e. The agar dilution assay to determine BC MIC was possibly not sensitive enough to detect phenotypic differences between the wild-type and mutant strains. Subsequently, growth curves for the mutant and the wild-type strains were obtained in the absence and presence of BC. The ∆*agrBDCA* mutant showed similar growth to EGD-e in BHI broth (Figure 2A). However, the growth of the ∆*agrBDCA* strain was completely inhibited when exposed to 2 μg/mL of BC (Figure 2B). These results support the hypothesis that the Agr system is involved in BC resistance. 

To further investigate the role of each component of the *agr* operon in BC resistance, the gene deletion mutants ∆*agrBD*, ∆*agrC,* and ∆*agrA* were constructed in this study. The mutants exhibited the same BC MICs as their parental strain. The results from growth curves demonstrated that the growth of the ∆*agrBD* and ∆*agrC* strains was impaired in the presence of BC compared to EGD-e (Figure 2B). Surprisingly, the ∆*agrA* strain showed better growth than the wild-type EGD-e, with shorter lag phase duration and higher maximum OD_600_ in BHI medium containing BC (Figure 2B). 

Complementation of the ∆*agrBDCA*, ∆*agrBD*, and ∆*agrC* strains partially restored the phenotype of the mutant strains under BC stress to the wild-type level (Figure 2C). The complemented strain C∆*agrA* completely restored growth in the presence of BC (Figure 2C). The deletion mutant containing pERL3 (the plasmid for complementation) was referred to as a vector control. As presented in Figure 2D, the vector controls exhibited a similar growth performance to their corresponding parental mutant strains. 

### 3.3. Both AgrBD and AgrC Are Essential for BC Resistance

Both AgrBD and AgrC contribute to BC resistance; however, the manner in which they function, individually or collectively, remains unclear. To clarify this point, the growth of the mutant strains ∆*agrBD*pERL3-*agrC* and ∆*agrC*pERL3-*agrBD* was measured. The growth curves for the mutant strains in the absence of BC are presented in Figure 2E. When exposed to BC, the constitutive expression of *agrC* did not improve the growth of the ∆*agrBD* mutant strain (Figure 2F). Similarly, the overexpression of *agrBD* did not influence the growth of the ∆*agrC* strain in the presence of BC (Figure 2F). Based on these findings, it appears that neither AgrBD nor AgrC can function alone in BC resistance. 

### 3.4. The RR Lmo1172 Plays a Role in BC Stress in the ∆agrA Strain

**Figure 3:**
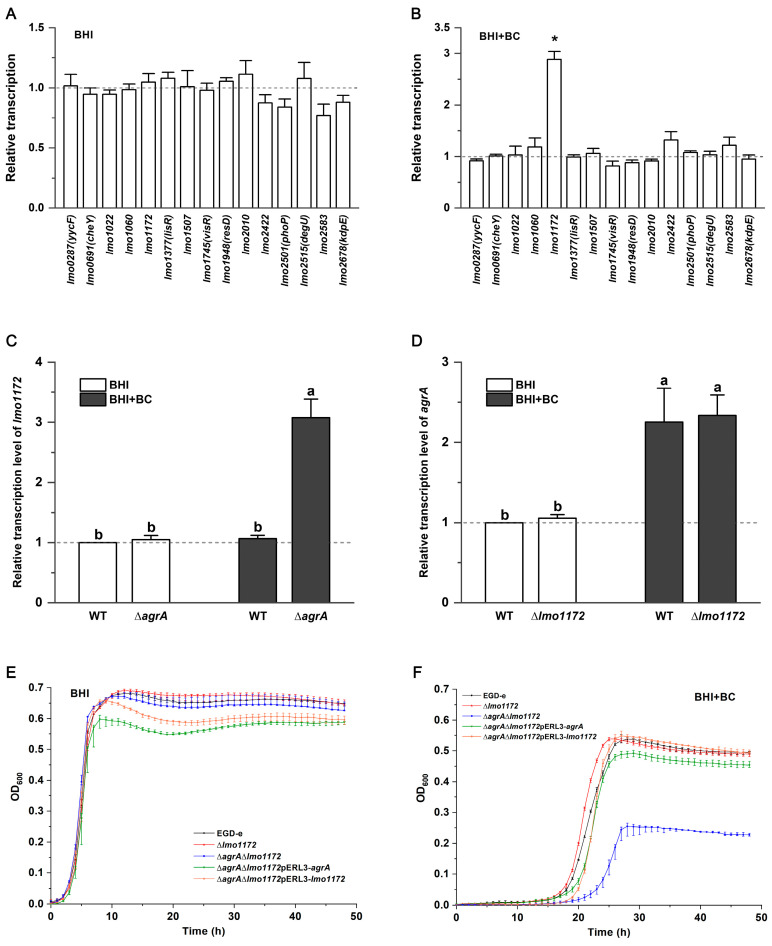
The RR Lmo1172 plays a role in BC stress in the ∆*agrA* strain. (**A**) Relative transcription levels of the other 15 RR-encoding genes in the ∆*agrA* strain grown in BHI broth. Results are presented as fold changes relative to the transcription level of the target gene in EGD-e. (**B**) Relative transcription levels of the other 15 RR-encoding genes in the ∆*agrA* strain grown in BHI broth with 2 μg/mL of BC. Results are presented as fold changes relative to the transcription level of the target gene in EGD-e with BC. The asterisk indicates a value that is statistically different from that of the control at *p* < 0.05. (**C**) Relative transcription levels of *lmo1172* in EGD-e and ∆*agrA* grown in BHI with or without BC. Results are presented as fold changes relative to the transcription level of *lmo1172* in EGD-e produced in BHI without BC. Bars labeled with different letters indicate significant differences at *p* < 0.05. (**D**) Relative transcription levels of *agrA* in EGD-e and ∆*lmo1172* grown in BHI with or without BC. Results are presented as fold changes relative to the transcription level of *agrA* in EGDe produced in BHI without BC. Bars labeled with different letters indicate significant differences at *p* < 0.05. Growth curves for *L. monocytogenes* wild-type EGD-e, ∆*lmo1172*, ∆*agrA*∆*lmo1172*, ∆*agrA*∆*lmo1172*pERL3-*agrA*, and ∆*agrA*∆*lmo1172*pERL3-*lmo1172* strains in (**E**) BHI broth and (**F**) BHI broth with 2 μg/mL of BC.

In the presence of BC, growth defects were observed in the mutant strain ∆*agrC*. However, the ∆*agrA* strain exhibited a better growth performance than the wild-type EGD-e. It was speculated that AgrC of *L. monocytogenes* could communicate with RRs other than AgrA to overcome the BC stress. The relative transcription levels of the other 15 RR-encoding genes in ∆*agrA* did not differ from those of EGD-e (Figure 3A). When exposed to BC, only the *lmo1172* gene was significantly upregulated (*p* < 0.05) in the ∆*agrA* strain (Figure 3B). The addition of BC, however, did not affect the transcription level of *lmo1172* in EGD-e (Figure 3C). On the other hand, there was no significant change in the transcription levels of *agrA* between EGD-e and the gene deletion mutant ∆*lmo1172,* and similar results were also observed in the presence of BC (Figure 3D). The data indicate that Lmo1172 could play a potential role in response to BC when the *agrA* gene is deleted. 

The results from growth curve analysis showed that a lack of *lmo1172* did not affect the bacterial growth in BHI with BC (Figure 3E,F), providing further evidence that Lmo1172 is not involved in BC stress when the *agrA* gene is present in the wild-type strain. However, the growth of the double deletion mutant ∆*agrA*∆*lmo1172* was restricted considerably, with a lower maximum OD_600_ under BC stress (Figure 3F). The constitutive expression of *agrA* or *lmo1172* restored the growth of the ∆*agrA*∆*lmo1172* mutant when exposed to BC (Figure 3F). These results suggest that Lmo1172 may be an alternative to AgrA in the phosphotransfer pathway. 

### 3.5. AgrC Cross-Phosphorylates the Noncognate RR Lmo1172 In Vitro

**Figure 4:**
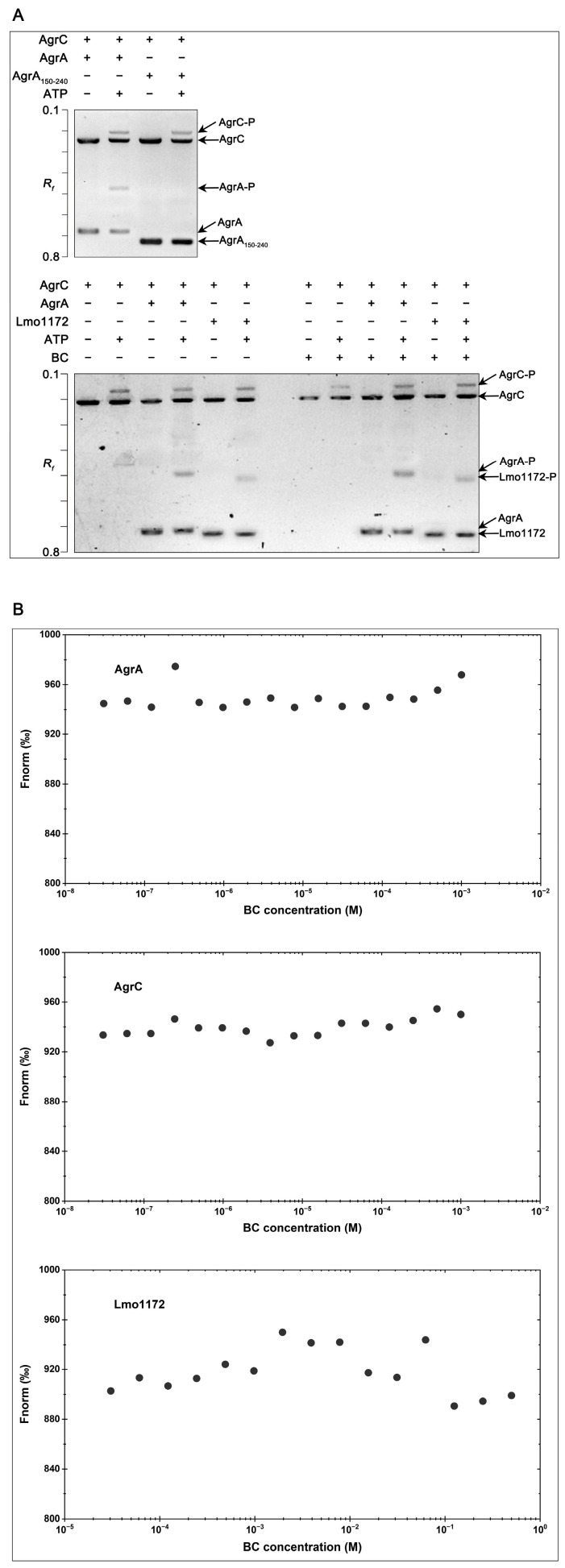
AgrC cross-phosphorylates the noncognate RR Lmo1172 in vitro. (**A**) Detection of AgrC autophosphorylation and phosphotransfer of AgrC with the RR AgrA and Lmo1172 by Phos-tag SDS-PAGE. The *R_f_* refers to the relative migration rate of proteins. The *R_f_* value of phosphorylated protein was smaller in comparison with that of the corresponding non-phosphorylated protein. The plus and minus symbols indicate the proteins/ATP/BC that are present/absent in each reaction. The molecular masses of AgrC, AgrA, AgrA_(150–242)_, and Lmo1172 were approximately 51, 30, 15, and 25 kDa, respectively. (**B**) Determination of interactions between BC and proteins by MST. Fnorm, normalized fluorescence.

To confirm the existence of cross-phosphorylation between the HK AgrC and the RR Lmo1172, the capacity of AgrC to phosphorylate Lmo1172 was tested in vitro. In this study, AgrC autophosphorylation and phosphotransfer of AgrC/AgrA and AgrC/Lmo1172 were assayed by Phos-tag SDS-PAGE. As shown in Figure 4A, an upshifted band corresponding to the autophosphorylated AgrC was observed when AgrC alone was incubated in the presence of ATP, showing that the purified AgrC kinase was active and capable of autophosphorylation. In a reaction containing AgrC and AgrA, a single upshifted band corresponding to the phosphorylated AgrA was observed as expected (Figure 4A), showing the occurrence of phosphotransfer from AgrC to AgrA. An upshifted band corresponding to phosphorylated Lmo1172 was also observed in the phosphotransfer reaction between AgrC and Lmo1172 (Figure 4A), suggesting that AgrC can phosphorylate Lmo1172 in vitro. Neither the AgrC nor AgrA phosphorylation level was affected by the addition of BC (Figure 4A). The results from microscale thermophoresis (MST) demonstrated that BC neither bound to AgrC nor AgrA (Figure 4B). It is likely that BC induces the Agr system via indirect action, rather than the direct interactions with AgrC and AgrA. The results also showed that BC had no effect on the phosphotransfer between AgrC and Lmo1172 (Figure 4A). Binding between BC and Lmo1172 was not observed by MST (Figure 4B). 

### 3.6. The Cognate HK Lmo1173 of Lmo1172 Is Not Involved in BC Stress

**Figure 5:**
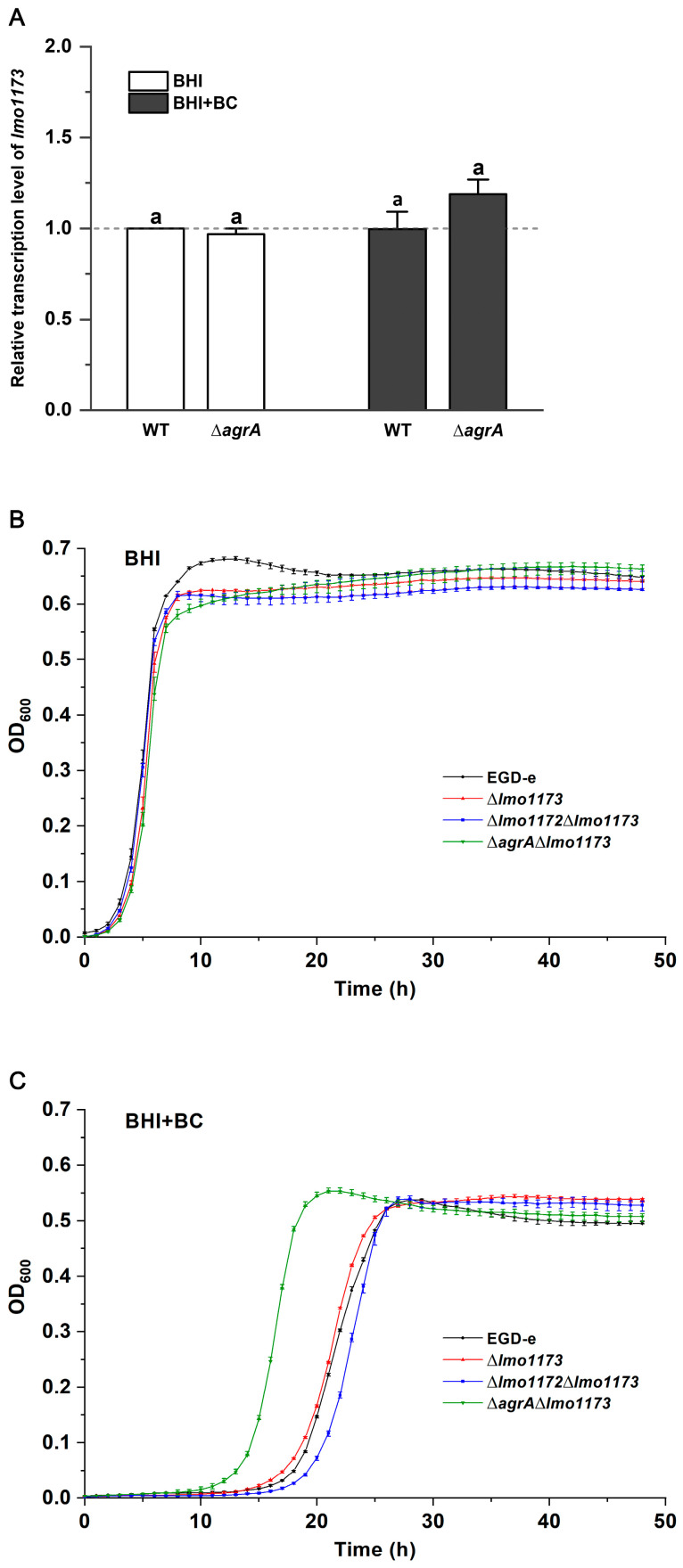
The cognate HK Lmo1173 of Lmo1172 is not involved in BC stress. (**A**) Relative transcription levels of *lmo1173* in EGD-e and ∆*agrA* grown in BHI with or without BC. Results are presented as fold changes relative to the transcription level of *lmo1173* in EGD-e produced in BHI without BC. Bars labeled with the same letter indicate significant differences at *p* > 0.05. Growth curves for *L. monocytogenes* wild-type EGD-e, ∆*lmo1173*, ∆*lmo1172*∆*lmo1173*, and ∆*agrA*∆*lmo1173* strains in (**B**) BHI broth and (**C**) BHI broth with 2 μg/mL of BC.

Results showed that no significant change in the transcription levels of *lmo1173* was observed when the wild-type strain EGD-e was exposed to BC (Figure 5A). The transcription levels of *lmo1173* were not affected by the deletion of *agrA* (Figure 5A). In the presence of BC, the transcription level of *lmo1173* in ∆*agrA* was similar to that in EGD-e (Figure 5A). These results suggest that the transcription of *lmo1173* in neither EGD-e nor the ∆*agrA* mutant is affected by BC. 

The single deletion mutant ∆*lmo1173* and the double deletion mutants ∆*lmo1172*∆*lmo1173* and ∆*agrA*∆*lmo1173* exhibited the same BC MICs as those of the wild-type strain. In the presence of BC, the growth of the ∆*lmo1173* and ∆*lmo1172*∆*lmo1173* strains was also similar to that of EGD-e; however, the growth curve of the ∆*agrA*∆*lmo1173* strain was similar to that of the ∆*agrA* mutant (Figure 5B,C). These pieces of evidence suggest that the HK Lmo1173 does not contribute to the response of *L. monocytogenes* to BC.

### 3.7. BC Is Not Sensed by the HK Lmo1173

**Figure 6:**
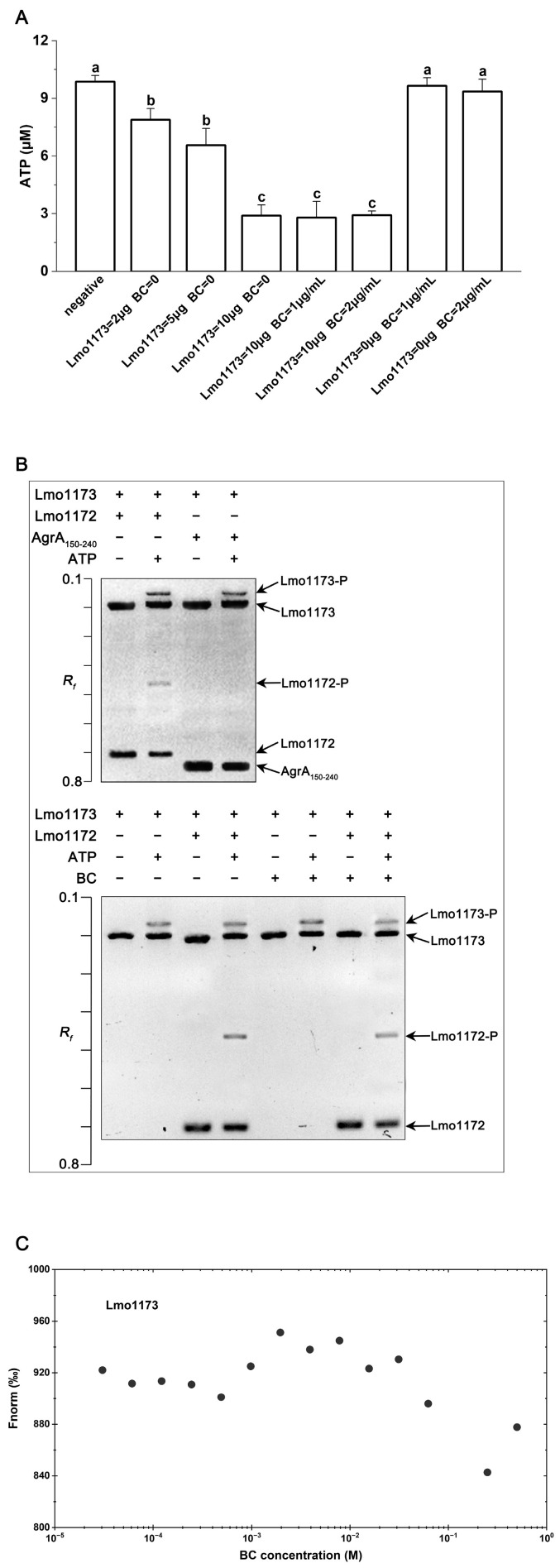
BC is not sensed by the HK Lmo1173. (**A**) The Lmo1173 kinase activity assay. Negative, ten μM ATP was added to the reaction system, and the kinase reaction was conducted without Lmo1173. Error bars represent the standard deviations of triplicate experiments (*n* = 3). Different letters on top of the bars represent significant differences (*p* < 0.05). (**B**) Detection of Lmo1173 autophosphorylation and phosphotransfer of Lmo1173 with the RR Lmo1172 by Phos-tag SDS-PAGE. The *R_f_* refers to the relative migration rate of proteins. The plus and minus symbols indicate the proteins/ATP/BC that are present/absent in each reaction. The molecular masses of Lmo1173, AgrA_(150–242)_, and Lmo1172 were approximately 57, 15, and 25 kDa, respectively. (**C**) Determination of interactions between BC and Lmo1173 by MST. Fnorm, normalized fluorescence.

In this study, the effects of BC on the autophosphorylation of the HK Lmo1173 were investigated by measuring the Lmo1173 kinase activity in vitro. Firstly, the Lmo1173 kinase activity was assayed using a commercial kit. The concentration of ATP in the reaction system decreased with the increase in incubation time, and the kinase activity showed a negative correlation with the residual amount of ATP after incubation. As presented in Figure 6A, the remaining ATP was decreased with increasing concentrations of Lmo1173, showing that the purified Lmo1173 was active. There was no significant change (*p* > 0.05) in the kinase activity of Lmo1173 in the presence of BC (Figure 6A). Next, Phos-tag SDS-PAGE was carried out to detect Lmo1173 autophosphorylation and phosphotransfer between Lmo1173 and Lmo1172. As shown in Figure 6B, Lmo1173 was able to autophosphorylate and phosphorylate Lmo1172. Furthermore, the addition of BC had no effect on Lmo1173’s self-phosphorylation and L1172’s phosphorylation (Figure 6B). 

MST was also performed to investigate the interaction between BC and Lmo1173 in vitro. The S-shaped binding curve was not observed, based on the MST data of BC and Lmo1173 (Figure 6C), indicating that BC could not bind to Lmo1173 in vitro. The results suggest that the HK Lmo1173 is not the sensor of BC. 

### 3.8. The Agr System Is Not Regulated by Lmo1172

**Figure 7:**
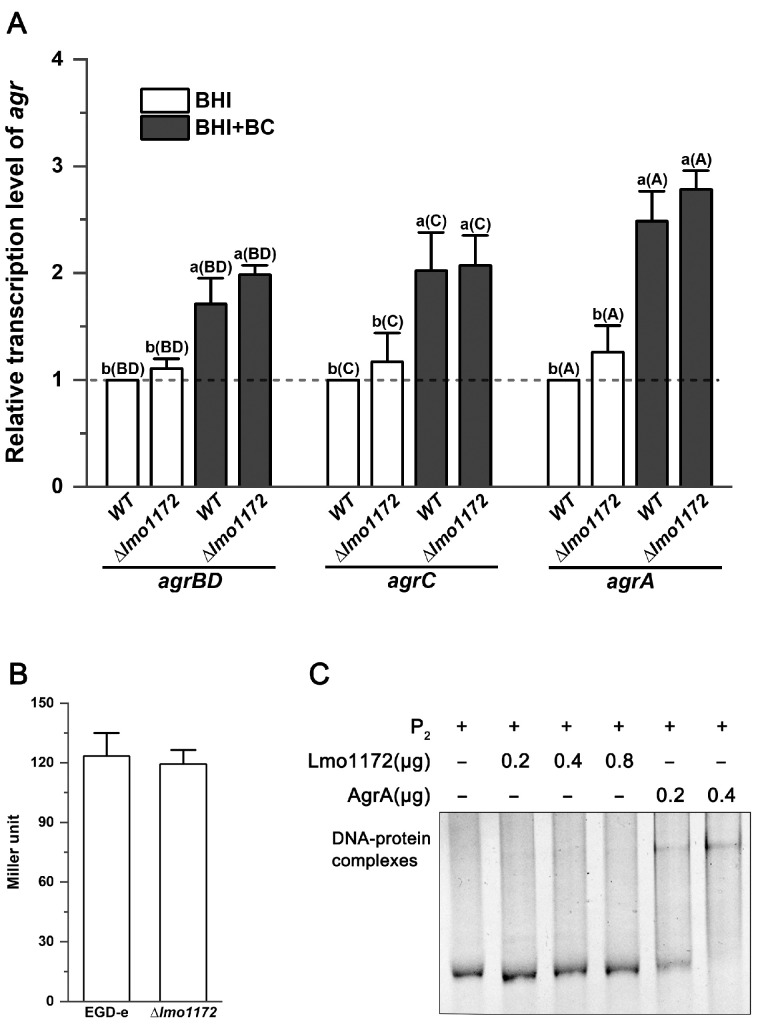
The Agr system is not regulated by Lmo1172. (**A**) Relative transcription levels of *agr* genes in EGD-e and ∆*lmo1172* grown in BHI with or without BC. Results are presented as fold changes relative to the transcription level of the target gene in EGD-e produced in BHI without BC. Different lowercase letters on top of the bars represent significant differences (*p* < 0.05). The capital letters in the brackets represent the *agr* gene; for example, BD represents *agrBD*. (**B**) Activity of the P_2_ promoter in EGD-e and the Δ*lmo1172* mutant strains grown in BHI broth. Error bars represent the standard deviation of triplicate experiments (*n* = 3). (**C**) EMSA for interaction between Lmo1172 and P_2_. AgrA was used as a positive control. The plus and minus symbols indicate the P_2_/proteins/BC that are present/absent in each reaction.

The Agr system is positively autoregulated by AgrA. Lmo1172 is regarded as a substitute for AgrA. Therefore, whether the *agr* operon is regulated by Lmo1172 was investigated. Results showed no significant difference in the transcription levels of the *agr* genes between EGD-e and the mutant strain ∆*lmo1172* (Figure 7A). Similar results were observed in the presence of BC (Figure 7A). Furthermore, the deletion of *lmo1172* had no effect on the activity of the *agr* promoter P_2_ (Figure 7B). Lmo1172 was incubated with the P_2_ promoter DNA for electrophoretic mobility assay (EMSA), but no shifted band of the protein–DNA complex was observed (Figure 7C), indicating that Lmo1172 was unable to bind to the P_2_ DNA sequence. These results suggest that Lmo1172 does not regulate the Agr system.

## 4. Discussion

The growth of the ∆*agr* deletion mutant strain was completely inhibited in relation to wild-type EGD-e under BC stress, indicating the critical role of the Agr system in response to BC. In the Agr system of *L. monocytogenes*, AgrB and AgrD are responsible for generating and secreting the AIP, and AgrC is the cognate transmembrane receptor of AIP [35]. The specific binding of AIP to AgrC leads to autophosphorylation of the cytoplasmic histidine kinase domain of AgrC [36,37]. The results demonstrated that both AgrBD and AgrC are essential for BC resistance in *L. monocytogenes*. In the ∆*agrBD* mutant, AgrC could not be activated by AIP. The ∆*agrC* mutant was able to produce AIP but lacked the receptor. 

Upon autophosphorylation of the HK AgrC, the RR AgrA catalyzes the phosphate group that is transferred from AgrC to reside in its own Asp [38,39]. This promotes the binding of AgrA to the P_2_ promoter and activates the transcription of the *agr* operon [17,18]. Although the Agr system is considered a global regulatory mechanism, it remains unclear which genes are regulated by AgrA. Riedel et al. [19] identified more than 650 differentially expressed genes by comparing the global gene expression profiles of an *agrD* deletion mutant and its parental strain EGD-e. These expression differences could be attributed to the low abundance of AgrA in the ∆*agrD* mutant or potential disruptions within a complex regulatory network. Unlike the ∆*agrC* strain, the growth of the ∆*agrA* mutant was better than that of the wild-type strain. Previous studies have reported similar findings, i.e., that an HK and its cognate RR of *L. monocytogenes* play different roles in the same phenotype or exhibit different growth under the same stress [40,41,42]. In the VirSR TCS, the RR VirR is required for the virulence of *L. monocytogenes*, but the HK VirS is not [41]. *L. monocytogenes* EGD-e can cope at low temperatures without the HK YycG, while the RR YycF is shown to be essential [40,42]. It has been speculated that crosstalk of TCSs could be one possible reason for this phenomenon [40,41,42]. TCSs have the potential for crosstalk, because large numbers of homologous signaling proteins are present in bacterial species [43,44]. It has been reported that many HKs can phosphorylate noncognate RR in vitro [45,46]. In this study, AgrC may communicate with another RR, leading to a better growth performance of the ∆*agrA* mutant.

In *L. monocytogenes* wild-type EGD-e, 16 TCSs have been identified, including 15 complete TCSs and an orphan RR. The results of this study showed that among the 15 RRs, other than AgrA, Lmo1172 could be the target of crosstalk from AgrC under BC stress. Regarding Lmo1172, there was limited understanding of its role in the stress response of *L. monocytogenes*. Chan et al. [40] reported the ∆*lmo1172* mutant strain to show reduced growth at 4 °C, suggesting that this gene is associated with the cold tolerance of *L. monocytogenes*. Phosphotransfer of AgrC/AgrA and AgrC/Lmo1172 in vitro was observed in this study, providing further evidence for crosstalk from AgrC to Lmo1172. In many cases, crosstalk has been detected solely when introducing diverse genetic perturbations, making it improbable to be present in the wild-type strain [44]. Consistently, this study showed that Lmo1172 was not involved in BC resistance in the wild-type EGD-e but played an important role in BC stress in the ∆*agrA* strain, indicating that the HK AgrC could crosstalk to the noncognate RR Lmo1172 to overcome BC stress only in the absence of AgrA. A previous study has reported that the presence of both RRs did not lead to crosstalk, indicating that the cognate RR normally out-competes the noncognate RR, thereby assisting in preventing crosstalk [44]. It was speculated that AgrC preferentially transfers the phosphate group to AgrA due to a higher affinity of AgrC for AgrA than for Lmo1172 in the wild-type EGD-e; when AgrA was deleted, AgrC-Lmo1172 became an alternative phosphotransfer pathway to cope with BC stress. However, further studies should be performed to confirm this hypothesis. 

Among the 15 RRs, only the *lmo1172* gene was significantly upregulated in the ∆*agrA* mutant strain when exposed to BC. Lmo1172 was thus selected as a potential target of crosstalk for AgrC. However, it is possible that AgrC can crosstalk to another RR without AgrA. In this study, Lmo1172 has been confirmed to be cross-phosphorylated by AgrC in vitro. Nevertheless, this might not reflect the true in vivo situation. Further studies are needed to detect the phosphorylation levels of proteins in vivo.

Our results showed that Lmo1173, the cognate HK of Lmo1172, was not involved in BC resistance in *L. monocytogenes*. Interaction between BC and Lmo1173 was not observed by MST. Furthermore, BC had no effect on the autophosphorylation level of Lmo1173. These suggest that Lmo1173 could not be activated by sensing BC. Therefore, the increased transcription of *lmo1172* in the ∆*agrA* mutant under BC stress was not due to activation of Lmo1173. 

Both transcription of the *agr* operon and the P_2_ promoter activity were found to be significantly upregulated in the presence of BC, suggesting that BC could induce transcription of the *agr* operon. However, the mechanism behind this upregulation is still not clear. Previous studies have reported that most inhibitors interfere with the Agr system by targeting AgrC or AgrA in *S. aureus* [47,48,49]. Our previous study has also found that cinnamaldehyde functions as a competitive inhibitor of AgrA on AgrA-P_2_, binding to inhibit transcription of the *agr* operon in *L. monocytogenes* [50]. Is it possible that AgrC or AgrA of *L. monocytogenes* is the target of BC? In this study, BC was neither bound to AgrC nor had any effect on the phosphorylation of AgrC in vitro. Similar results were also observed for AgrA. These findings eliminated the possibility of interactions between BC and the AgrCA TCS. Therefore, it is believed that BC indirectly induced transcription of the Agr system.

It has been found that transcription of the *agr* genes was lower in the ∆*agrA* in-frame deletion mutant compared to the parental strain EGD-e, suggesting that the Agr system is autoregulated [19]. In our previous study, specific binding of AgrA and the Agr promoter P_2_ was observed, which supports the positive regulation of AgrA on the Agr system [32]. Additionally, MouR, a novel virulence regulator, has been confirmed to control the transcription of the Agr system in *L. monocytogenes* by directly binding to the P_2_ promoter [50]. In this study, the finding that the Agr system is not regulated by Lmo1172 indicates that Lmo1172 does not become a part of the Agr system when AgrA is absent. It was assumed that among the genes that are directly or indirectly regulated by Lmo1172, certain gene(s) could be associated with BC resistance. Further studies should be performed to determine the gene(s) that are controlled by Lmo1172, which is/are necessary for BC resistance in *L. monocytogenes*. 

## 5. Conclusions

To summarize, BC indirectly induces transcription of the Agr system in *L. monocytogenes* by an unknown mechanism. Both AgrBD and AgrC are essential for BC resistance. When AgrA is absent, crosstalk from AgrC to its noncognate RR Lmo1172 plays a role in response to BC.

## Data Availability

Data will be made available on request.

## Supplementary Materials

The following supporting information can be downloaded at https://www.mdpi.com/article/10.3390/microorganisms12020392/s1: Table S1: Bacterial strains and plasmids used in this study; Table S2: Primers used in this study.
