# Supplementary material for: Cross-Phosphorylation between AgrC Histidine Kinase and the Noncognate Response Regulator Lmo1172 in Listeria monocytogenes under Benzalkonium Chloride Stress
Source: Microorganisms. 2024 Feb 16;12(2):392. doi: 10.3390/microorganisms12020392 (PMC10891604; doi:10.3390/microorganisms12020392)
Supplement: Supplementary file 1 [file microorganisms-12-00392-s001.zip › Table S1-R2.pdf]

Table S1. Bacterial strains and plasmids used in this study.

Strain or Plasmid	Genotype[a]	Reference or Source
Strain		
L. monocytogenes		
EGD-e	Wild-type strain; deposition number ATCC® BAA-679™	A gift from Qin Luo
EGD-epPTPL-P_2	EGD-e containing pPTPL-P_2	49
Δ*agrBDCA*	In-frame deletion of *agrBDCA*	21
Δ*agrBD*	In-frame deletion of *agrBD*	This study
Δ*agrC*	In-frame deletion of *agrC*	This study
Δ*agrA*	In-frame deletion of *agrA*	49
CΔ*agrBDCA*	Complemented strain of Δ*agrBDCA*	This study
CΔ*agrBD*	Complemented strain of Δ*agrBD*	This study
CΔ*agrC*	Complemented strain of Δ*agrC*	This study
CΔ*agrA*	Complemented strain of Δ*agrA*	49
Δ*agrBDCA*pERL3	Δ*agrBDCA* containing pERL3	This study
Δ*agrBD*pERL3	Δ*agrBD* containing pERL3	This study
Δ*agrC*pERL3	Δ*agrC* containing pERL3	This study
Δ*agrA*pERL3	Δ*agrA* containing pERL3	49
Δ*agrBD*pERL3-*agrC*	Δ*agrBD* containing pERL3-*agrC*	This study
Δ*agrC*pERL3-*agrBD*	Δ*agrC* containing pERL3-*agrBD*	This study
Δ*lmo1172*	In-frame deletion of *lmo1172*	This study
Δ*agrA*Δ*lmo1172*	In-frame deletion of *agrA* and *lmo1172*	This study
Δ*agrA*Δ*lmo1172*pERL3-*agrA*	Δ*agrA*Δ*lmo1172* containing pERL3-*agrA*	This study
Δ*agrA*Δ*lmo1172*pERL3-*lmo1172*	Δ*agrA*Δ*lmo1172* containing pERL3-*lmo1172*	This study
Δ*lmo1173*	In-frame deletion of *lmo1173*	This study
Δ*lmo1172*Δ*lmo1173*	In-frame deletion of *lmo1172* and *lmo1173*	This study

Δ*agrA*Δ*lmo1173*	In-frame deletion of *agrA* and *lmo1173*	This study
Δ*lmo1172*pPTPL-P_2	Δ*lmo1172* containing pPTPL-P_2	This study
E. coli		
DH5α	Chemical competent strain	Biomed, Beijing, China
BL21(DE3)	Expression host	Biomed, Beijing, China
C43(DE3)	Expression host	Biomed, Beijing, China
Plasmid		
pPTPL-P_2	pPTPL containing the putative promoter region of the *agr* operon	49
pMAD	Cloning shuttle integration vector plasmid, Amp^R and Ery^R	27
pMAD-Δ*agrBD*	pMAD containing homologous arms up- and downstream of EGD-e *agrBD*	This study
pMAD-Δ*agrC*	pMAD containing homologous arms up- and downstream of EGD-e *agrC*	This study
pERL3	Plasmid capable of replication in *L. monocytogenes*, Ery^R	28
pERL3-*agrBDCA*	pERL3 containing the upstream region and coding sequence of *agrBDCA*	This study
pERL3-*agrBD*	pERL3 containing the promoter region and coding sequence of *agrBD*	This study
pERL3-*agrC*	pERL3 containing the promoter region and coding sequence of *agrC*	This study
pERL3-*agrA*	pERL3 containing the promoter region and coding sequence of *agrA*	49
pERL3-*lmo1172*	pERL3 containing the upstream region and coding sequence of *lmo1172*	This study
pET28a	Expression vector, Amp^R	Biomed, Beijing, China
pET32a-*lmo1172*	pET28a containing the full length of *lmo1172*	This study
pET28a-*lmo1173*	pET28a containing the full length of *lmo1173*	This study

[a] Amp^R: resistance to ampicillin; Ery^R: resistance to erythromycin.
