# Supplementary material for: Cross-Phosphorylation between AgrC Histidine Kinase and the Noncognate Response Regulator Lmo1172 in Listeria monocytogenes under Benzalkonium Chloride Stress
Source: Microorganisms. 2024 Feb 16;12(2):392. doi: 10.3390/microorganisms12020392 (PMC10891604; doi:10.3390/microorganisms12020392)
Supplement: Supplementary file 1 [file microorganisms-12-00392-s001.zip › Table S2-R2.pdf]

Table S2. Primers used in this study.

Gene/Protein	Primer Name	Sequence (5'-3')[a]	Annealing Temperature	Reference
RT-qPCR				
agrBD	RTlmo0048-1	GCTGGAAAGATGATGAAGAA	55℃	22
	RTlmo0048-2	TCTATCGGTCACTTTCGTATCT		
agrC	RTlmo0050-1	CTCTACAAAAGGAGAAGGTCGT	55℃	22
	RTlmo0050-2	TCTATCGGTCACTTTCGTATCT		
agrA	RTlmo0051-1	GCAAGCAGAAGAACGGATTT	57℃	22
	RTlmo0051-2	CTGTGGCACCGATAAAATGA		
lmo0287	RTlmo0287-1	CTTGGTGCGGATGACTATGT	56℃	This study
	RTlmo0287-2	TCTTCCTCGGCTGTGCTT		
lmo0691	RTlmo0691-1	ATGCGATGTTCATGCGTAC	58℃	This study
	RTlmo0691-2	TGGCGTCTACAACCATACCT		
lmo1022	RTlmo1022-1	GGCACTTGAAGCTGGAGCG	58℃	This study
	RTlmo1022-2	TTTAGCGGTTAGCCGTTGC		
lmo1060	RTlmo1060-1	TACCGAAACCTTTGAAGTGAT	54℃	This study
	RTlmo1060-2	ATGCGTATTGATAATGGACGT		
lmo1172	RTlmo1172-1	CTTACCGCATTTAGTGACCC	55℃	This study
	RTlmo1172-2	TTTCTTGTTTCTCGCCCTTT		
lmo1377	RTlmo1377-1	AGCGGTTGCTAATGATGGA	58℃	This study
	RTlmo1377-2	AGCGAAAGGTTTGACGATG		
lmo1507	RTlmo1507-1	TGATATTGCGATTATTGACC	53℃	This study
	RTlmo1507-2	CCATTAAAGTAAGTGGGCTA		
lmo1745	RTlmo1745-1	TAATGGCGAGGATAAATGC	54℃	This study
	RTlmo1745-2	CTTTCATCTTCCCAAAGTGC		

lmo1948	RTlmo1948-1	TGATGATGCCTGGTAAAGA	51°C	This study
	RTlmo1948-2	GTGGGAAGGTAATAATGTCTC		
lmo2010	RTlmo2010-1	ATTGAGGGATTGGAAGAGG	54°C	This study
	RTlmo2010-2	CTGCGTTAATATGGAAGTCGT		
lmo2422	RTlmo2422-1	GTGCCAAGGCTGAAGACAA	56°C	This study
	RTlmo2422-2	GCCACCAACCGTTACAACA		
lmo2501	RTlmo2501-1	TGACCTAACACCGAAAGAA	52°C	This study
	RTlmo2501-2	CAAATGGCTAACATGAAGC		
lmo2515	RTlmo2515-1	GCACTTAGAGCTGGAGCA	52°C	This study
	RTlmo2515-2	TAGCCATAAACACCTTGAGA		
lmo2583	RTlmo2583-1	TGCCTAACATGGATGGTTT	55°C	This study
	RTlmo2583-2	TTTGACTTCGCTTGCTTGA		
lmo2678	RTlmo2678-1	AAAAGTGCCGATTATTGTTG	55°C	This study
	RTlmo2678-2	GAGGAGTTCCGATGTGCC		
lmo1173	RTlmo1173-1	TCATTCATCATACACCACCAA	55°C	This study
	RTlmo1173-2	TTAGGACGGCAATAACTCG		
16S rRNA	RT16S1	GGGAGGCAGCAGTAGGGA	55°C	29
	RT16S2	CCGTCAAGGGACAAGCAG		
Mutant strain construction				
agrBD	lmo0048/9-1	*NNNNNN*<u>GGATC</u>CGTCTACAAAGTTGATGGGATT (*Bam*HI)	50°C	This study
	lmo0048/9-2	ACTCATAGAAGAATCCGCAAATCATCTTTCCAGCGGTC		
	lmo0048/9-3	GACCGCTGGAAAGATGATTTGCGGATTCTTCTATGAGT	51°C	
	lmo0048/9-4	*NNNNNN*<u>ACGCG</u>TGCTAAGACAGTAAGAGCAACG (*Mlu*I)		
agrC	lmo0050-1	*NNNNNN*<u>GGATC</u>AGAAAAGCGATGATAGGGA (*Bam*HI)	53°C	This study
	lmo0050-2	CATAAATTCATCCCCATTCTTTGCTATAGTAACCAATCCCTCT		
	lmo0050-3	AGAGGGATTGGTTACTATAGCAAAGAATGGGGATGAATTTATG	53°C	

	lmo0050-4	*NNNNNN*ACGCGACCTGTGGCACCGATAAA (*Mlu*I)		
lmo1172	lmo1172-1	*NNNNNN*GGATCCATAATGCG TCTTGTCTTG (*Bam*HI)	55℃	This study
	lmo1172-2	AACACATTTCTCGAATCGTTGTCCTCTTCTTCTGCTATTGAT		
	lmo1172-3	ATCAATAGCAGAAGAAGAGGACAACGATTCGAGAAATGTGTT	55℃	
	lmo1172-4	*NNNNNN*ACGCGTTCCTGCTCGTCCTCTTC (*Mlu*I)		
lmo1173	lmo1173-1	*NNNNNN*GGATCAATAGAACCGAGCACAAAGAC (*Bam*HI)	54℃	This study
	lmo1173-2	GCTTCATCGCTTTTTCATAGCGCTCATCACAATCGTTTC		
	lmo1173-3	GAAACGATT GTGATGAGCGCTATGAAAAAGCGATGAAGC	54℃	
	lmo1173-4	*NNNNNN*ACGCGTTCTTGTTTCACGGTCTACTTT (*Mlu*I)		
lmo1172lmo1173	lmo1172/3-1	*NNNNNN*GGATCCATAATGCG TCTTGTCTTG (*Bam*HI)	54℃	This study
	lmo1172/3-2	GCTTCATCGCTTTTTCATAGGTCCTCTTCTTCTGCTATTGAT		
	lmo1172/3-3	ATCAATAGCAGAAGAAGAGGACCTATGAAAAAGCGATGAAGC	54℃	
	lmo1172/3-4	*NNNNNN*ACGCGTTCTTGTTTCACGGTCTACTTT (*Mlu*I)		
Complementation				
agrBDCA	lmo0048/0051-5	*NNNNNN*GTCGACTAAAATGGAAAAGCCAACTG (*Sal*I)	50℃	This study
	lmo0048/0051-6	*NNNNNN*GGATCCTACCTTTTGCCTGAATTTGA (*Bam*HI)		
agrBD	lmo0048/9-5	*NNNNNN*GAGCTCTAAAATGGAAAAGCCAACTG (*Sac*I)	52℃	This study
	lmo0048/9-6	*NNNNNN*GGATCCGAAGAATCCGCAACTTTCA (*Bam*HI)		
lmo1172	lmo1172-5	*NNNNNN*GAGCTCATAGAACCGAGCACAAAGAC (*Sac*I)	50℃	This study
	lmo1172-6	*NNNNNN*GGATCCTTAGTCATCGCTCATCACAA (*Bam*HI)		
Expression of protein				
Lmo1172	lmo1172-7	*NNNNNN*GGATCCGTGACAGGAATGAATGGAAG (*Bam*HI)	55℃	This study
	lmo1172-8	*NNNNNN*CTCGAGGCTTCTTCTAAGATGTCTCGG (*Xho*I)		
Lmo1173	lmo1173-7	*NNNNNN*GGATCCATGACTAAAACGATTCGAGA (*Bam*HI)	55℃	This study
	lmo1173-8	*NNNNNN*CTCGAGCTACTTCTTTGTAGCATGGATA (*Xho*I)		
EMSA				

P_2	P-3	TAAAATGGAAAAGCCAACTG	50℃	49
	P-4	CTAATTCACCTCCACTAATA		

[a] Restriction sites are underlined. N, any of the bases.
